# Small cardamom genome: development and utilization of microsatellite markers from a draft genome sequence of *Elettaria cardamomum* Maton

**DOI:** 10.3389/fpls.2023.1161499

**Published:** 2023-05-10

**Authors:** Ambika Baldev Gaikwad, Ratna Kumari, Sheel Yadav, Parimalan Rangan, Dhammaprakash Pandhari Wankhede, KV. Bhat

**Affiliations:** Division of Genomic Resources, Indian Council of Agriculture Research-National Bureau of Plant Genetic Resources, New Delhi, India

**Keywords:** small cardamom, whole genome sequence (WGS), simple sequence repeats (SSRs), allele, genetic diversity, microsatellite marker database

## Abstract

Small cardamom (*Elettaria cardamomum* Maton), the queen of spices, is the third most expensive spice in the world after saffron and vanilla, valued highly for its aroma and taste. This perennial herbaceous plant is a native of coastal parts of Southern India and displays a significant amount of morphological diversity. Its genetic potential has not been exploited due to lack of genomic resources limiting our understanding of the genome and important metabolic pathways which give it the economic advantage in the spice industry. Here, we report upon the *de novo* assembled, draft whole genome sequence of cardamom variety, Njallani Green Gold. We used a hybrid assembly strategy using the reads from the Oxford Nanopore, Illumina and 10x Genomics GemCode sequencing chemistries. The assembled genome length was 1.06 Gb (gigabases) which is close to the estimated genome size of cardamom. More than 75% of the genome was captured in 8000 scaffolds with a N50 of 0.15 Mb. The genome appears to have a high repeat content and 68055 gene models were predicted. The genome is close to *Musa* species and displays an expansion and contraction in different gene families. The draft assembly was used for *in silico* mining of simple sequence repeats (SSRs). A total of 2,50,571 SSRs were identified of which 2,18,270 were perfect SSRs and 32,301 were compound SSRs. Among the perfect SSRs, trinucleotides were most abundant (1,25,329) and hexanucleotide repeats appear least (2,380). From the 2,50,571 SSRs mined, 2,27,808 primer pairs were designed based on flanking sequence information. Wet lab validation was performed for 246 SSR loci and based on their amplification profiles, 60 SSR markers were used for diversity analysis of a set of 60 diverse cardamom accessions. The average number of alleles detected per locus were 14.57 with a minimum of 4 and maximum of 30 alleles. Population structure analysis revealed the presence of high degree of admixtures which could primarily be due to cross-pollination prevalent in this species. The SSR markers identified would help in the development of gene or trait-linked markers which can be subsequently used for marker-assisted breeding for crop improvement in cardamom. The information on utilization of the SSR loci for generation of markers has been developed into a public database, ‘cardamomSSRdb’ that is freely available for use by the cardamom community.

## Introduction

Small cardamom (*Elettaria cardamomum* Maton) is a monocot species belonging to the family Zingiberaceae with 2n=4x=48.This species is believed to have originated in the rainforests of the Western Ghats of South India (Kuriakose et al., 2009; Hrideek et al., 2011; Nadukeri et al., 2020a, b). As it is a shade loving plant, it is mainly grown under canopies which provides suitable environment for plant growth. In India, it is majorly grown in the three southern states of Kerala, Karnataka and Tamil Nadu, with Kerala alone accounting for more than 58% of the annual production (Horticultural Statistics at a glance, 2018). Owing to its pleasant taste and aroma, cardamom is commonly referred to as the ‘Queen of spices’. The seeds and fruits of cardamom are the economically important parts and have found wide applicability across the culinary and traditional medicine spectrum (Verma et al., 2009; Kaliyaperumal et al., 2020). The medicinal properties of cardamom are attributed to the essential oil which is extracted from its fruits. This oil possesses antibacterial, anti-inflammatory and antispasmodic activities (al-Zuhair et al., 1996; Sobral et al., 2014; Alam et al., 2021).

The Zingiberaceae family comprises of more than 1500 species (Li et al., 2021). Most species of this family are valued as natural sources of spices, perfumes, herbal medicines, dyes, etc (Kumar et al., 2013).The molecular studies for species delineation within the family have so far relied on the use of the nuclear internal transcribed spacer (ITS) based loci and traditional chloroplast markers (Kress et al., 2002; Ngamriabsakul et al., 2000). The availability of chloroplast genomes of eighteen different species belonging to four different genera of Zingiberaceae is a valuable resource in order to understand the evolutionary dynamics within the family (Li et al., 2021). However, the lack of whole genome sequences (WGS) limits marker identification and utilization. So far, the WGS information is available for only two Zingiberaceae species i.e., *Curcuma longa* (Chakraborty et al., 2021; Yin et al., 2022) and *Curcuma alismatifolia* (Dong et al., 2022). The scarcity of genomic resources in this family creates a bottleneck for carrying out future studies which are aimed at crop improvement. Therefore, the generation of comprehensive genomic resources is of paramount importance for deriving insights into species characterization and phylogenetic diversity. In small cardamom, RAPD (Random Amplified Polymorphic DNA) markers have been used previously for molecular characterization of genotypes (Radhakrishnan and Mohanan, 2005). ISSR (Inter Simple Sequence Repeat) markers have been also been used to assess the genetic variability among accessions which included wild collections, landraces, feral and released varieties (Jose et al., 2014; Anjali et al., 2016). The development of other marker systems which are sequence based will expand the scope and reliability for conducting genetic diversity analysis. Simple sequence repeats (SSR) or microsatellite markers, are one of the most widely used markers for genetic diversity analysis (Cavagnaro et al., 2010; Wang et al., 2010; Feng et al., 2016; Ali et al., 2019; Kumari et al., 2019; Kapoor et al., 2020; Li et al., 2022). Their relative abundance, multi- allelic nature, co-dominance, high levels of reproducibility and ease with which these can be scored, are some of the reasons for their widespread utility across a wide range a species. Furthermore, they are present across both the coding and non-coding regions of the genome and can be used for cross-species amplification in related species and genera. In cardamom, previously, SSR markers have been developed using selective hybridization enrichment method and these were subsequently used for diversity and cross transferability studies (Cyriac et al., 2016). However, these were very few in number (140). Also, a few EST-SSRs (200 in number) have been developed using the EST sequences from *Curcuma longa* and used for diversity analysis in cardamom (Anjali et al., 2015). It is important to identify a comprehensive set of SSR markers which would provide genome-wide coverage. This would facilitate comparative and functional genomics studies for cardamom in the future.

Here, we report the WGS of one of the most popular, high-yielding varieties of cardamom, Njallani Green Gold. The genome has been assembled using long and short reads and an exhaustive number of SSRs were identified. A select set was then used to genotype a collection of accessions for the purpose of validation and subsequently a database was developed for ease of access and utilization. This will serve as a rich source of genomic resources in small cardamom.

## Materials and methods

### Genome sequencing and assembly

**Figure 1:**
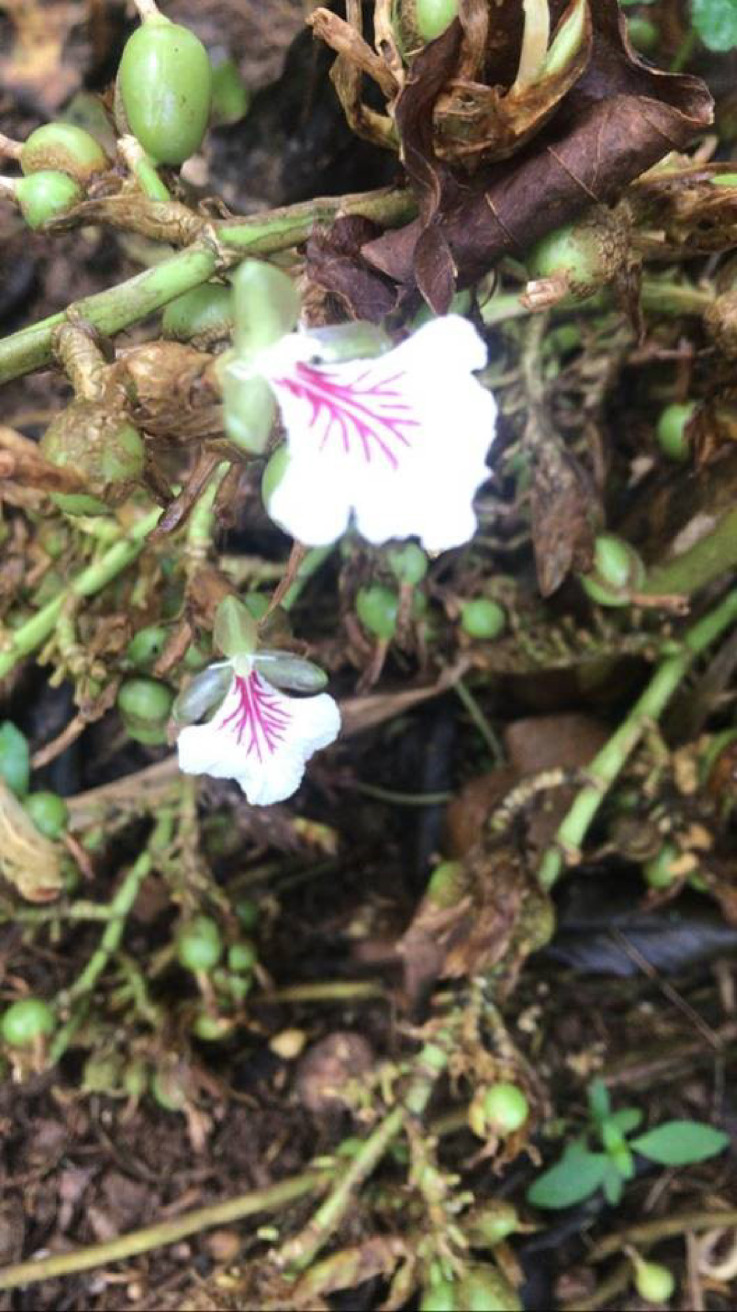
The flowers and capsules of small cardamom (*Elettaria cardamomum* Maton).

Total genomic DNA was isolated from the leaves of the popular farmer’s variety ‘Njallani Green Gold’ (**Figure 1**) using the CTAB extraction method (Doyle and Doyle, 1987). The purified DNA was checked on 0.8% agarose gel and quantified on NanoDrop (DS-11 spectrophotometer, DeNovix, Wilmington, Delaware). Illumina (paired –end and 10X chromium) and Oxford Nanopore genomic libraries were prepared as per the manufacture’s protocol and sequenced on Illumina HiSeq X Ten sequencer and Oxford Nanopore P24 PromethION sequencing platforms, respectively. The genome size was evaluated by k-mer (k=16) distribution analysis with Jellyfish using the Illumina paired-end reads (105.11 Gb). The Bioinformatics pipeline was as follows. The Nanopore data correction was carried out using Canu (version 1.6). The corrected Nanopore data was further used for *de novo* assembly using Wtdbg2 (https://github.com/ruanjue/wtdbg2). This assembly was further polished using Pilon (Walker et al., 2014. BWA (version 0.7.17) Mem algorithm was used to map the Illumina data on assembly obtained using Wtdbg2. The 10X chromium data along with Nanopore assembly was used by ARCS (https://github.com/bcgsc/arcs) for further scaffolding.

### Repeat masking

Tandem repeat finder was utilized to identify tandem repeats in polished genome. In brief, *de novo* repeat library was generated using RepeatModeler version 2.0.1 (http://www.repeatmasker.org/RepeatModeler/) to predict transposable elements in the unannotated genome assemblies. Three, *de novo* repeat-finding programs, RECON version 1.08 (Bao and Eddy, 2002), RepeatScout version 1.0.5 and LtrHarvest (genometools-1.5.9)/Ltr_retriever (version 2.9.0) were employed to identify the boundaries of repetitive elements and to build consensus models of interspersed repeats. RepeatMasker version 4.1.0 (https://www.repeatmasker.org/) was used to annotate DNA sequences for interspersed repeats and identification of low complexity DNA sequences.

### BUSCO genome assessment

We selected Benchmarking Universal Single-Copy Orthologs (BUSCOs; version 3, http://busco.ezlab.org embryophyta_odb10) to assess the completeness of the assembled genome against *Arabidopsis thaliana* species with 1375 Plant BUSCOs.

### Gene prediction

Seqping (https://sourceforge.net/projects/seqping/, version 0.1.45.1) was used for gene prediction of the masked sequence. It is an automated pipeline that performs gene prediction using self trained HMM (hidden Markov model) models and transcriptomic data. The program processes the genome and transcriptome sequences of a target species through Glimmer HMM, SNAP, and AUGUSTUS training pipeline that ends with the program MAKER2 combining the predictions from the three models in association with the transcriptomic evidence. The pipeline generated species-specific HMMs and is able to predict genes that are not biased to other model organisms. Genes were clustered using CDHIT v4.6 (http://cd-hit.org) software at a sequence similarity of 90%.

### Gene annotation

Genes were mapped against NR database (updated May 2020) using blastx (NCBI-2.2.29+) and Plant Transcription factor database (http://planttfdb.cbi.pku.edu.cn/, version 5.0). Gene annotation was done using Uniprot database and KEGG (Kyoto Encyclopedia of Genes and Genomes) database (https://www.genome.jp/kegg/pathway.html). GO visualisation was done using WEGO version 2.0.

### Phylogenetic analysis

BUSCO was used for phylogenomics. BUSCO was done for all the 14 species to predict conserved genes. *Amborella trichopoda, Ananas comosus*, *Arabidopsis thaliana*, *Cocus nucifera*, *Elettaria cardamomum*, *Glycine max*, *Musa acuminata*, *Musa balbisiana*, *Oryza sativa, Phoenix dactylifera*, *Populus deltoides*, *Solanum lycopersicum*, *Sorghum bicolor* and *Vitis vinifera.* Complete BUSCO genes were extracted from all the genomes. Alignment of the peptide sequences of genes was done using MUSCLE (version 3.8.31) program and analysed using MEGAX (https://www.megasoftware.net/). UPGMA (Unweighted Pair Group Method with Arithmetic Mean) method was used for constructing phylogenetic tree.

### *In silico* mining of microsatellite repeats

The assembled genome sequence was searched for presence of different microsatellite (di to hexa nucleotide) simple repeats as well as complex repeats following the default parameter of MISA -MIcroSAtellite identification tool (http://pgrc.ipk-gatersleben.de/misa/).The SSRs were identified from the draft genome using MISA perl scripts (Beier et al., 2017). The search criteria included minimum of six repeats of dinucleotides, minimum five repeats for trinucleotides, tetranucleotides, pentanucleotides and hexanucleotides.

### Development and validation of SSR primers

The SSR primers were designed from the flanking sequences of SSRs identified in the genomic sequences of cardamom by using software Primer 3 (Rozen and Skaletsky, 2000; Untergasser et al., 2012). Primers were designed for 2,27,808 of the 2,50,571 SSRs identified. Out of the 2,27,808 SSR primers, 246 primers were synthesized for wet lab validation (**Supplementary Table 1**). PCR was carried out in a total volume of 20 µl comprising of 1X PCR buffer, 2.5mM MgCl_2_, 1µM primer, 0.2mM of each dNTPs, 1U Taq DNA polymerase (NEB) and 15 ng template DNA in thermal cycler (Eppendorf). The amplification conditions were as follows: Initial denaturation at 95°C for 5 min followed by 35 cycles of denaturation at 95°C for 1 min, annealing at 44-52°C for 1 min and extension at 72°C for 1 min followed by final extension at 72°C for 10 mins.

### Genetic diversity analysis

The plant material included 60 accessions (**Table 1**) of *Elettaria cardamomum* collected from Karnataka and Kerala (Nadukeri et al., 2020b). DNA was isolated from the leaves using the CTAB extraction method and quantified on the Nanodrop spectrophotometer (DS-11 spectrophotometer, DeNovix, Wilmington, Delaware). Genetic diversity was analysed by amplification at 60 SSR loci (**Table 2**). The amplified products were resolved on QIAxcel multi-capillary system using QIAxcel High Resolution Kit 1200 (QIAGEN, No. 929002, New Delhi, Qiagen India Pvt. Ltd.), 50-800 bp v2.0 Qx DNA size marker (QIAGEN, No. 929561) and 15bp/1000 bp QX alignment marker (QIAGEN, No. 929521). PCR products were separated with high resolution run method OM700 with a sample injection time of 10 seconds. The allelic sizes of each sample were resolved and calculated in the form of gel profiles and peaks using QIAxcel ScreenGel software (QIAGEN, v1.5). The SSR amplification products were scored across the lanes according to their molecular weight. The polymorphism information content (PIC) for each pair of SSR primers was calculated using the formula:

**Table 1:** List of cardamom accessions used for genetic diversity analysis (as per Nadukeri et al., 2020b).

S.No.	Genotype	Origin of genotype	Morphological attributes
1	CL692	ZAHRS Mudigere	Prostrate panicle, oval/oblong in shape, bold, good yield
2	CL688	ZAHRS Mudigere	Prostrate panicle, bold capsules
3	Wayanad	Wayanad, Kerala	Thick stem from the base
4	CL671	ZAHRS Mudigere	Prostrate panicle
5	CL622	ZAHRS Mudigere	Medium bold capsules with pale green colour
6	D751	Mudigere, Karnataka	Medium bold capsules with pale green colour
7	PV4	Pampadumpara	Prostrate panicle, forms a part of the Walayar collection
8	D168	Mudigere, Karnataka	Semi-erect panicle
9	PDP4	Pampadumpara	Erect panicle and medium plant height
10	PDP14	Pampadumpara	Erect panicle and medium plant height
11	Pink Pseudostem	Myladumpara	Prostrate panicle and pink coloured pseudo stem
12	CL73	ZAHRS, Mudigere	Prostrate panicle, short span of flowering
13	M1	ZAHRS, Mudigere(Local check)	Prostrate panicle, tolerant to thrips and shoot borer, pubescent leaves
14	M2	ZAHRS, Mudigere (Local check)	Prostrate panicle,Clonal selection from CL-683
15	Dharmada	Sri Lanka	Erect panicle, green colour capsule
16	Ceylone-5	Periyar Tiger reserve, Kerala	Erect panicle and good plant height
17	P10	Pampadumpara	Round capsules with medium plant height
18	PDP12	Pampadumpara	Semi-erect panicle, late and long flowering span
19	K5	Kalmane, Mudigere, Karnataka	Semi-erect panicle, late flowering
20	D516	Mudigere, Karnataka	Semi-erect panicle, round oblong capsules
21	KMRD2	Kammardi, KoppaTaluk, Karnataka	Semi-erect panicle, green colour capsules with good plant height
22	D11	Mudigere, Karnataka	Semi-erect panicle, green capsules
23	D140	Mudigere, Karnataka	Semi-erect, green capsules
24	KMRD10	Kammardi, KoppaTaluk, Karnataka	Semi-erect, green colour capsules with good plant height
25	P1	Pampadumpara	Short panicle, early maturing variety with slightly ribbed light green capsules.
26	CL679	ZAHRS Mudigere	Bold capsules, panicle length is good
27	GreenGold	Vandanmedu, Idukki	Semi-erect panicle, dark green coloured bold capsules, robust in nature, perform well under intensive cultivation
28	HS1	Handi selection, Mudigere	Prostrate panicle, drought tolerant and good yield, round capsules
29	MCC61	Myladumpara	Erect panicle, bold capsules and produce more tillers
30	MCC12	Myladumpara	Erect panicle, terminal flowering
31	MCC21	Myladumpara	Erect panicle, bold capsules and produce more tillers
32	CSS-800	IISR, Appangala	Semi-erect panicle, bold, dark deep green capsule
33	CSS-872	IISR, Appangala	High yielding and dark green capsules
34	ICRI-2	Myladumpara	Erect panicle, clonal selection from MCC-61
35	SKP21	ICRI, Sakleshpur	Prostrate panicle, early and short span of flowering
36	SKP72	ICRI, Sakleshpur	Prostrate panicle, early and short span of flowering, good plant height
37	CL-698	ZAHRS, Mudigere	Bold capsules, panicle length is good.
38	MCC18	Myladumpara	Bold capsules, number of bearing suckers are more
39	CL-668	ZAHRS Mudigere	Medium bold capsules with green in colour
40	CCS800	IISR, Appangala	High yielding and dark green capsules
41	SEL-98	ZAHRS, Mudigere	Bold capsules, panicle length is good.
42	10-6-D10	ZAHRS, Mudigere	Superior for number of bearing suckers and high yielding
43	2-5-D11	ZAHRS, Mudigere	Superior for number of bearing suckers, high yield and medium bold capsules
44	12-7-D11	ZAHRS, Mudigere	Good plant height with more number of bearing suckers
45	26-16-D11	ZAHRS, Mudigere	Medium oval shape capsules
46	29-9-D11	ZAHRS, Mudigere	Medium bold oval shape capsules
47	S-1	Pampadumpara	Semi-erect panicle
48	MHC-73	Myladumpara	Medium plant height, with pale yellow capsules
49	MHC-26	Myladumpara	Erect panicle, oval shape capsules
50	MCC-309	Myladumpara	Erect to semi-erect panicle, oval shape capsules
51	APG-284	IISR, Appangala	Good yield, bold capsule and dark green colour leaves
52	APG-293	IISR, Appangala	Good yield and bold capsule
53	MHC-10	Myladumpara	Prostrate panicle, medium plant height with pale yellow capsules
54	SKP-170	ICRI, Sakleshpur	Semi-erect panicle, with round to oblong capsules
55	MHC-18	Myladumpara	Erect panicle, a hybrid of the germplasm collections, deep green, angular bold capsules, performance is fairly good even under rainfed conditions
56	MHC-13	Myladumpara	Prostrate panicle, medium plant height, with green to pale yellow capsules
57	SKP-165	ICRI, Sakleshpur	Good plant height with more number of bearing suckers
58	PS-44	Pampadumpara	Bold capsules, good panicle length.
59	MCC-200	Myladumpara	Early flowering type
60	CCS-1	IISR, Appangala	Maximum bearing and bold capsules

**Table 2:** List of 60 SSR primer pairs used for diversity analysis in 60 accessions of cardamom.

S. No.	Primer ID	Repeat type	Forward primer sequence	Reverse primer sequence	No. of alleles	Allele size (bp)
1	EC1	(TG)20	CTTTGGTTGTATAGGAAATG	TATGCTGCTAACTCTTTCTC	5	338
2	EC4	(TG)18	ATAAATTAGAATTCGCTGTG	TATGGCCTTGTAATAGATGT	4	225
3	EC5	(TG)17	ACTTGTCCGTGATACTTATG	AGAAGCAAAGGAAGAATATC	7	315
4	EC7	(TG)16	CTTGGATGTAGTTTCACAAT	CTCTCAATGCTAATTCCTAA	4	300
5	EC8	(TG)15	ATACAAACCATTAAAACCAA	AACAATATTGGCACATAGTC	4	123
6	EC12	(TG)14	TGTAAATATCCTTTTCTGGA	TGTCAAGGATACTTTCTCAC	7	241
7	EC14	(TG)14	ATTCATTTGAACATAAGCAC	AGCATGATATTACACCAAAC	8	269
8	EC24	(TC)18	TAAGTTTGTATTTTTGAGGG	CTCTACATCGTCTATGCAAT	11	304
9	EC25	(TC)17	GGAGTGATAAAACACTACCA	CTCGTATTGTTTCAGAAAAG	7	240
10	EC28	(TC)17	GTTCTCGTAATTTCAATGTC	CCAATCAAACTTCATAAAAC	9	273
11	EC29	(TC)16	ATACAGAGTAGGCAATTGAA	GCTTGAGAATACTACCAAAA	6	349
12	EC33	(TC)15	ATATTAATGTTTGCTAACCG	AAATCACAAAGCAAAATAAG	8	340
13	EC34	(TC)15	ATTGACTATTTCATGTTTGC	TAAGAAACCAAAATAGATGG	13	289
14	EC35	(TC)14	ATTTATACTATCGCTGTTGC	GAAACGAGAAAGAGGAAG	7	206
15	EC37	(TC)14	TGACAGCGTATGTGTATTTA	AAATCATATTCATTCAAACC	14	313
16	EC41	(TC)14	CAAGTTACTTCTTCGGTTTA	CCAGCTTTTGAGATTAGATA	8	274
17	EC43	(TC)14	TTTCTAGTTCATTATCTCCG	AGTTCACTAGCTATCTCCCT	17	308
18	EC46	(TC)14	TATGGTTAATTGCATTCTCT	TGTTCTAACATCTTGAATCC	30	298
19	EC47	(TC)14	TAGATGAAGACTGAGAATGC	CGACAAAAGAAAGTACAGAC	18	271
20	EC48	(TC)14	CCAAACTTTTTATTATCCAC	ACATTGAAGTGTGTTGAGAT	8	176
21	EC49	(TC)14	TGAACTTCAATTTCTTGTTT	TTTGTTTATCGGACTTCTTA	13	333
22	EC83	(GT)17	GTACATATGGGTTGATTGAC	ACAAGCTAATGATCTTGGTA	11	306
23	EC86	(GT)13	GCATGATACCACTAACAAAT	AGAGTGTTGCAAAATAAAAG	16	208
24	EC89	(GA)18	GAAAATCAGTGGATTGATAA	AGATTTTGGAATCTCTCTTT	26	342
25	EC90	(GA)18	GTGCTACGCTGTACTTTTAT	TTAAGGAATAGCTGAAATTG	19	341
26	EC92	(GA)17	TCTAATGATAGCTTGATCGT	AGTAGCTGATACAGAATTGC	26	219
27	EC93	(GA)16	AAATGCTTCCATATAACTCA	TAATTGCAAAGAGGAATAAG	16	296
28	EC95	(GA)16	TCTTTCTTTCTCCCTAGAAT	TGTTTCAATCAAATACATCA	13	253
29	EC96	(GA)16	AAGCCCATCTAATTAAAGTT	CTGTCCTTTGTTTACTCTTG	12	336
30	EC102	(GA)15	GATATGATGATCCTTTTGTG	TGTTCTACTATTTAGCTGCC	9	138
31	EC104	(GA)14	CTACTTGGCTACCCTGTT	CTGCCGTAGAAGTAAATAAG	12	258
32	EC106	(GA)14	AACTTTCTTGTGATGAAAAA	CTGTTAGAAGTATTCGCATT	19	260
33	EC124	(CT)17	GTGCAGAAAAGAACAATAAT	AATACCAAAGCTCATCTACA	24	272
34	EC130	(CT)15	ATTATAGTAATTACCGCTGC	CTTTATCGATGACAAGGTC	15	314
35	EC132	(CT)15	GTGGTGTTTTCGAGAGTAA	AAGAAGAAGAAGGACAAAAT	20	311
36	EC140	(CT)14	CATTTAGGATAGTCTTTTGC	CTGACATTAAACGAATCAAT	15	120
37	EC141	(CT)14	GTAAATGTCAATTGGAAAAG	CTTCTAACGTGACAAAAACT	20	173
38	EC142	(CT)14	AACAAAATGAGTCAAACAAC	AACATGAGAAATGACAGAAG	20	199
39	EC145	(CT)14	TTTCTCACTGAGGACTAGC	CTGGTAATAATGGGTAATTG	15	306
40	EC168	(AG)18	ACTAAACGAGTGATACCTGA	CTCTCCCTCAATTTCTTAAT	17	190
41	EC169	(AG)17	CCACGACTTTAATCTACTTG	AGAAACCAAGAAAAGAACTC	26	299
42	EC172	(AG)17	ATCAAAGCATACTCTCTCAA	AGAGATTTTCTTCCTGTTTT	15	207
43	EC181	(AG)15	ATGAATGGTGTTTAGAGTTG	TCAATGATGCATAACAATTA	26	327
44	EC182	(AG)15	ATATACTAGTGAACCACGGA	CAAAATCTCTCCTTTTCTCT	16	313
45	EC183	(AG)15	TAATGTAATCATCAATGCAA	TGAGTTTTACTGTCCAGAAC	22	158
46	EC187	(AG)14	GAATCTTGTATGATGCTTGT	TGTATCCGTTCAAATAACTT	24	298
47	EC192	(AG)14	TGTTCCTAGAAGCATAACAT	TTGCTTCCTAAAATCTATTG	22	322
48	EC193	(AG)14	CAAAAACGACCTAGTTAAAG	GACGATCAATTAAACCTCTA	17	222
49	EC195	(AG)14	ATCAAATAAATGGAATGAAA	AAATTTCAGGTCTTACCG	12	339
50	EC196	(AG)14	AAATATTAAAAACACGTTCG	TTCAGACTTTGAACTCAACT	11	186
51	EC197	(AG)14	TTAATTCTCCCATTGTCTAA	AATTACATTATGGCTTGAG	15	280
52	EC212	(AC)18	GAATAACCATCTGTCAGAAA	ACAAACGATAGAAGAAATTG	9	347
53	EC232	(TCT)14	ACCAATTGAAATGTGATTAG	CGAAAATACTTATTGTCCAC	11	244
54	EC234	(GAA)14	GAGAATTGCCTTTTGTAATA	CTTTCCACATAGTTCAGTGT	18	195
55	EC235	(GAA)13	GCTATTGTACGGAAGATAAA	AGATCAAACGCTGTAATTT	18	289
56	EC236	(CTT)15	AGATTGAACACTAATTGGAA	GAAGAAGATGATGATGAGAA	15	305
57	EC240	(AGA)13	CCAATTCACTTGGTTATATT	ATTAATGAAGATGAAGCAGA	17	269
58	EC242	(AAT)11	CCTAAGTGAGAATAGCTTCA	AATTATCTCTCTTCCCAGAT	17	240
59	EC243	(AAG)13	AACAAAAGGGAGAAAGTTAT	TGATCTACCAGTATTTCCAC	17	257
60	EC245	(AAG)11	TACCTTCGTACTTCCAATAG	GTAGATGAAATCCCAGTACA	13	282


$${\text{PIC}}_{j}=1-\sum_{i=1}^{\text{n}}\text{P}i^{2}$$

where *i* is *i*-th allele of the *j*-th marker, *n* is the number of the *j*-th marker’s alleles, Р is the allele frequency (Botstein et al., 1980). The data was analyzed using Polysat package of R (Clark and Jasieniuk, 2011) choosing Bruvo distance (Bruvo et al., 2004) for generating distance matrix and dendrogram was generated using hierarchical clustering.

### Population structure

The SSR allelic data on 60 accessions was used to run the software STRUCTURE version 2.3.4 (Pritchard et al., 2000). In order to determine the correct K value, the run parameters were set as Length of burn-in 10,000, Number of MCMC after burn-ins at 50000 and number of iterations at 20. The result data was used to estimate the delta (k) values and these were plotted against the K values to obtain a biplot to determine the correct K as 5 following Evanno method (Earl and vonHoldt, 2012). Following inference of correct K as 5, the analysis was done to determine the allelic affinities of each putative panicle type groups using the parameters, Length of burn-in 500,000, Number of MCMC after burn-ins at 7,50,000 and number of iterations at 20. The results presented are based on this final analysis. Based on panicle type, cardamom can be classified into the Malabar (prostrate), Mysore (erect) and Vazhukka (semi-erect or intermediate) types. Unambiguous data on panicle type was available for only 39 of the 60 accessions used in this study (**Table 1**). Accordingly, there were 15, 10 and 14 accessions under the three panicle type groups, Malabar (prostrate), Mysore (erect) and Vazhukka (semi-erect), respectively and hence only these 39 accessions were used for the analyses of population sub- structure.

### Development of microsatellite marker database

*Elettaria cardamomum* microsatellite database (CardamomSSRdb) is an interactive and relational online database that contains comprehensive information on small cardamom genomic SSRs that were identified using Misa script and primers designed using Primer 3 software. It is based on “three tier level schema architecture and organization” with client, server and database. PHP has been used to design the dynamic, interactive and user-friendly interface of the database and My SQL server for storing the genomic SSR data in tabulated form. Other than genomic SSR data and statistics, the database also contains step by step pictorial tutorial to facilitate hassle free usage by the user.

## Results and discussion

### Genome sequencing and assembly

The cardamom genome was sequenced using a hybrid sequencing approach which included the long read chemistry from the Oxford Nanopore, Illumina short read data and 10x Genomics GemCode linked read data (**Table 3**). Similar strategy involving the use of more than one sequencing chemistries has been employed for whole genome sequencing of *Curcuma longa* and *Curcuma alismatifolia*, both belonging to the Zingiberaceae family (Chakraborty et al., 2021; Dong et al., 2022; Yin et al., 2022) and also in other species(Hu et al., 2019; Wang et al., 2019). The Nanopore sequencing generated a total data of 141.90 Gb. The average read length obtained was 28 Kb. After data correction, the reads were assembled *de novo* and an assembled sequence of 1.06 Gb was generated. Based on K-mer depth distribution analysis the total length of the genome was estimated to be ~ 1.3 Gb (**Supplementary Table 2**). A genome sequencing depth of 108.46 x with more than 75% of the genome was captured in the assembled sequence. The assembled genome was polished using the Illumina paired-end (PE) data. The Illumina sequencing chemistry generated a total of 105.11 Gb data. The assembly of the reads resulted in generation of 30,000 contigs. The minimum contig length increased more than four times from 3419 bp to 12736 bp after polishing. The 10 X GemCode sequencing data along with the Nanopore *de novo* assembly was used for scaffolding. The final assembly consisted of 8000 scaffolds with a scaffold N50 of 0.15 Mb (**Table 4**). The assembled genome length was 1.06 Gb with 39.36% GC content. Previously, the genome size for cardamom has been estimated to be 1.4 Gb based on flow-cytometry analysis with a 2C nuclear DNA of 2.87 pg (Anjali et al., 2016). Our assembled genome length of 1.06 Gb reveals that we were able to sequence > 75% of the predicted genome length. The draft genome assembled in the present study can provide a base to build on for any future refinements in the genome assembly in terms of reducing the gaps present. The assembly quality in terms of completeness was estimated using BUSCO (Benchmarking Universal Single-Copy Orthologs) and more than 72% of the genes were found to be complete (C) with more than 99% single-copy genes, on analysis with 1,375 total BUSCO groups for *Arabidopsis thaliana* (**Supplementary Table 3**).

**Table 3:** Metrics of Nanopore, Illumina and 10 X GemCode Sequencing data.

Nanopore		Illumina		10 X GemCode	
Number of reads	13964122	Number of reads	696152122	Number of reads	612562452
Total data	141.90 Gb	Total data	105.11 Gb	Total data	92.4 Gb
Mean Read Length	28 Kb	Read Length	151 bp	Read Length	151 bp
Largest Read Length	969 Kb	Number of contigs	13145	Number of scaffolds	8000
Number of contigs	30638	Minimum contig length	12736 bp		
Minimum contig length	3419 bp				

**Table 4:** Cardamom genome assembly and annotation.

Assembly features	
Length of genome	1.06 Gb
Number of scaffolds	8000
N50	0.15 Mb
Longest scaffold	3.9 Mb
Smallest scaffold	33.3 Kb
Mean length	0.13 Mb
GC content (%)	39.36
Number of genes	68,055
Number of tRNA genes	478
Number of sRNA genes	1852

### Genome annotation

Genome annotation led to identification of 68,055 gene models. The non-protein coding genes included 478 tRNA genes and 1852 other small RNA genes. The repetitive sequences constituted 71% of the assembled genome (**Table 5**), with retrotransposons contributing to as high as 47% of the repeats. Amongst the retrotansposons the LTR retrotansposons accounted for 46% of the assembled sequence with ~ 36% of Copia and ~ 9% of Gypsy elements. The tandem repeats in the form of satellites (satDNA) constituted 0.11% of the genome with 1223 satellite elements identified.

**Table 5:** Organization of repetitive sequences in the cardamom genome.

Repeats	Length	In genome (%)	Number of elements
Total interspersed repeats	759115910	71.15	–
Retrotransposons	504982486	47.33	324440
DNA transposons	10371139	0.97	11829

A total of 38,400 genes were annotated (**Supplementary Table 4**) and 14,731 genes were mapped to the Plant Transcription factor database (**Supplementary Table 5**). The enrichment analysis of the genes identified in the cardamom genome was performed through the Gene Ontology (GO) and KEGG enrichment analysis. The GO analysis revealed that the genes were enriched in the GO terms of cellular component, molecular function and biological process. Amongst the GO category of cellular component, the GO term membrane (GO: 0016020) was most abundant. Similarly for the GO category of molecular function, the GO term, GO: 16390, binding was most abundant. For biological process GO category, the GO terms cellular process (GO: 0009987) and metabolic process (GO: 0008152) were most represented (**Supplementary Table 6** and **Supplementary Figure 1**). The KEGG enrichment analysis revealed an enrichment of genes involved in metabolic pathways and biosynthesis of secondary metabolites (**Supplementary Table 7** and **Supplementary Figure 2**). The species belonging to the Zingiberaceae family are known to be rich sources of secondary metabolites which have found immense utility in field of traditional medicine, identification of genes involved in biosynthesis of these compounds would help in understanding the genetic basis of secondary metabolite production in cardamom (Vairappan et al., 2013; Ghasemzadeh et al., 2016).

### Comparative genomic analysis

**Figure 2:**
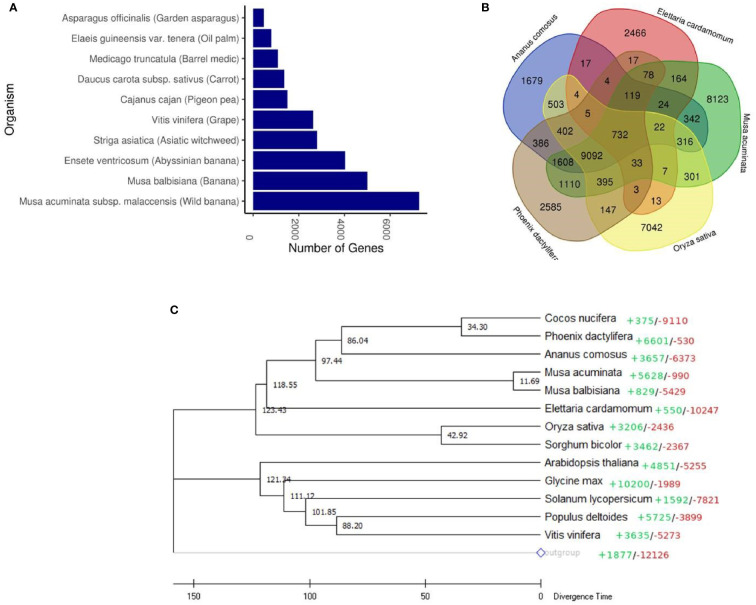
Comparative genomic analysis of cardamom (*Elettaria cardamomum* Maton). **(A)** Orthologous genes found in different plant species. **(B)** Venn diagram representing the clusters of gene families in cardamom shared with *Musa acuminata*, *Oryza sativa*, *Phoenix dactylifera* and *Ananas comosus*. **(C)** Phylogenetic tree of cardamom with 13 other species based on the single-copy protein sequences. Amborella trichopoda was used as an outgroup species. The colored figures represent CAFÉ-based estimates of gene family expansions (+) and contractions (-). The scale at the bottom depicts the divergence time in million years ago (Mya) with individual figures given at the branches.

In order to gain an insight into cardamom evolution, we compared the genome of cardamom with other species. Highest similarities were observed with the wild banana species, *Musa acuminata* with 7,265 othologous genes identified, followed by the banana species, *Musa balbisiana* with 4,996 orthologous genes (**Figure 2A** and **Supplementary Table 8**). A total of 3,708 gene families were identified in cardamom. The conservation of these gene families was examined amongst four different species that included *Musa acuminata* (Family: Musaceae), *Oryza sativa* (Family: Poaceae), *Phoenix dactylifera* (Family: Arecaceae) and *Ananas comosus* (Family: Bromeliaceae). It was observed that highest number of gene families, 31% (1179) were conserved between cardamom and *Musa acuminata.* This is in agreement with the taxonomic classification, as both *Musa acuminata* and cardamom belong to the same order of Zingiberales (D’Hont et al., 2012). A total of 732 gene families were conserved amongst all the five species with 2,466 gene families specific to cardamom (**Figure 2B**).

A phylogenetic tree was generated based on the single-copy protein sequences identified in cardamom and thirteen other species. These species were selected based on their taxonomic classification with representation from diverse taxonomic groups. Amongst the monocotyledons (Commelinids), we selected *Sorghum bicolour, Oryza sativa*, *Ananas comosus*, *Elettaria cardamomum*, *Musa acuminata, Musa balbisiana, Phoenix dactylifera*, *Cocos nucifera* and amongst the eudicots, *Arabidopsis thaliana*, *Glycine max*, *Solanum lycopersicum*, *Populus deltoides* and *Vitis vinifera* were selected (**Figure 2C**). The basal angiosperm *Amborella trichopoda* was used as an outgroup species. The phylogenetic analysis clearly separated the Commelinids from the eudicots. It was observed that the genome of cardamom was most similar to the genomes of *Musa acuminata and Musa balbisiana.* This is in corroboration with the previous studies where it has been observed that the Zingiberaceae species like *C. longa*, *Z. officinale* and *C. alismatifolia* are phylogenetically closer to the Musaceae family species, *M. acuminate* and *Musa balbisiana* (D’Hont et al., 2012; Dong et al., 2022). The time of divergence of Musaceae from the Zingiberaceae (*Elettaria cardamomum*) was estimated to be ~80 Mya (million years ago). Additionally, we observed that the Arecales (*Phoenix dactylifera*, *Cocos nucifera)* are more closely related to Zingiberales (including *Musa*) than to Poales, as has been reported previously (D’Hont et al., 2012; Yin et al., 2022). However, amongst the poales, *Ananas comosus* was observed to be placed closer to Arecales than Poales. The earlier divergence of *Ananas comosus* from the Poales might be a reason for this observation (Qian and Jin, 2016). Another plausible reason for this could be the genome assembly of pineapple used in the reported analysis. The assembly used is the MD2 v1 assembly (Ming et al., 2015) which is not at the chromosome level and has lesser number of genes predicted and annotated, than the newer version of the MD2 assembly (Yow et al., 2021). The CAFE based analysis of gene family expansion and contraction revealed that 550 gene families show expansion in cardamom and 10247 gene families were contracted.

### Identification and characterization of SSRs from the cardamom genome

**Figure 3:**
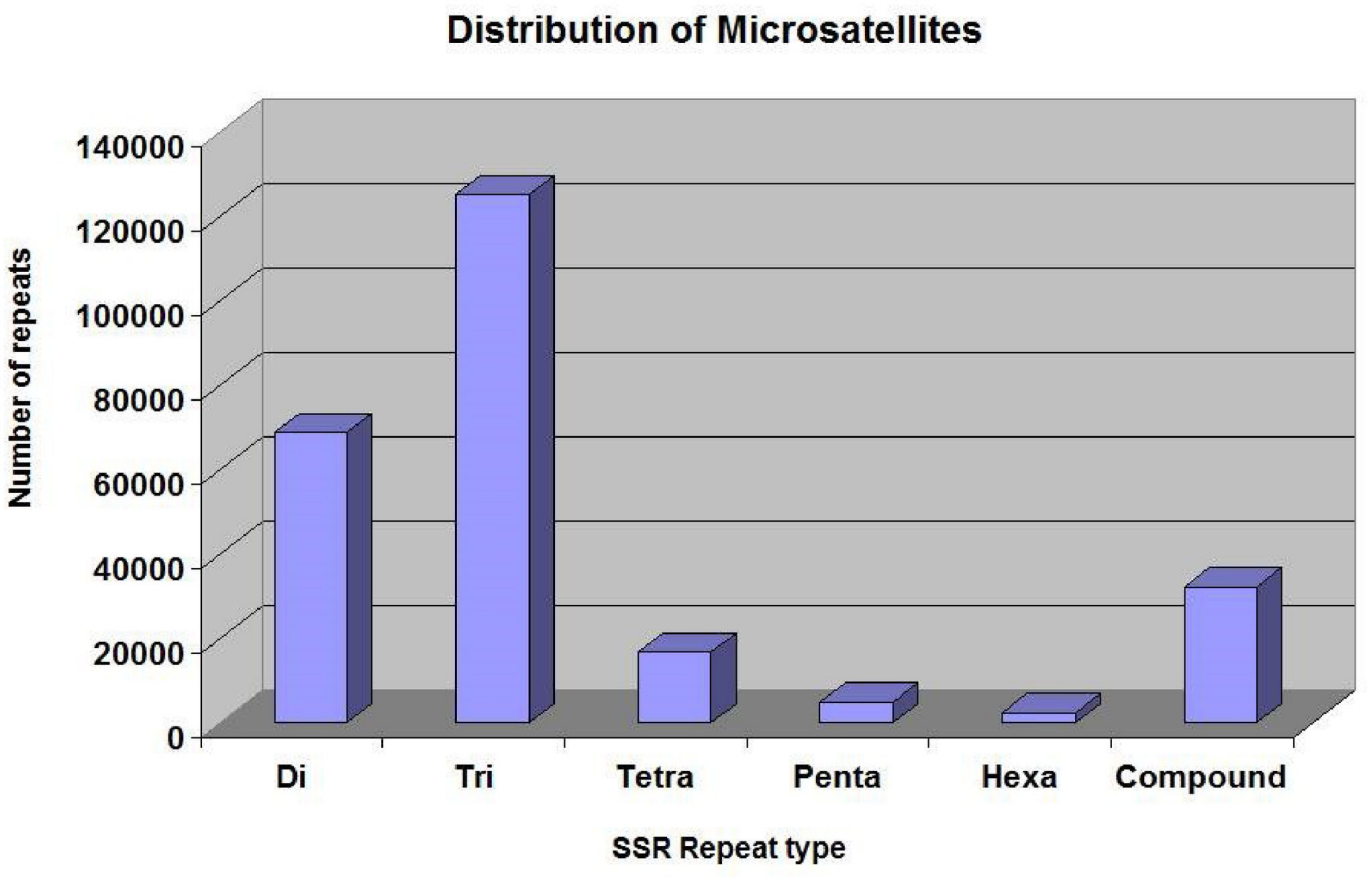
The distribution of different types of SSRs in the cardamom genome.

**Figure 4:**
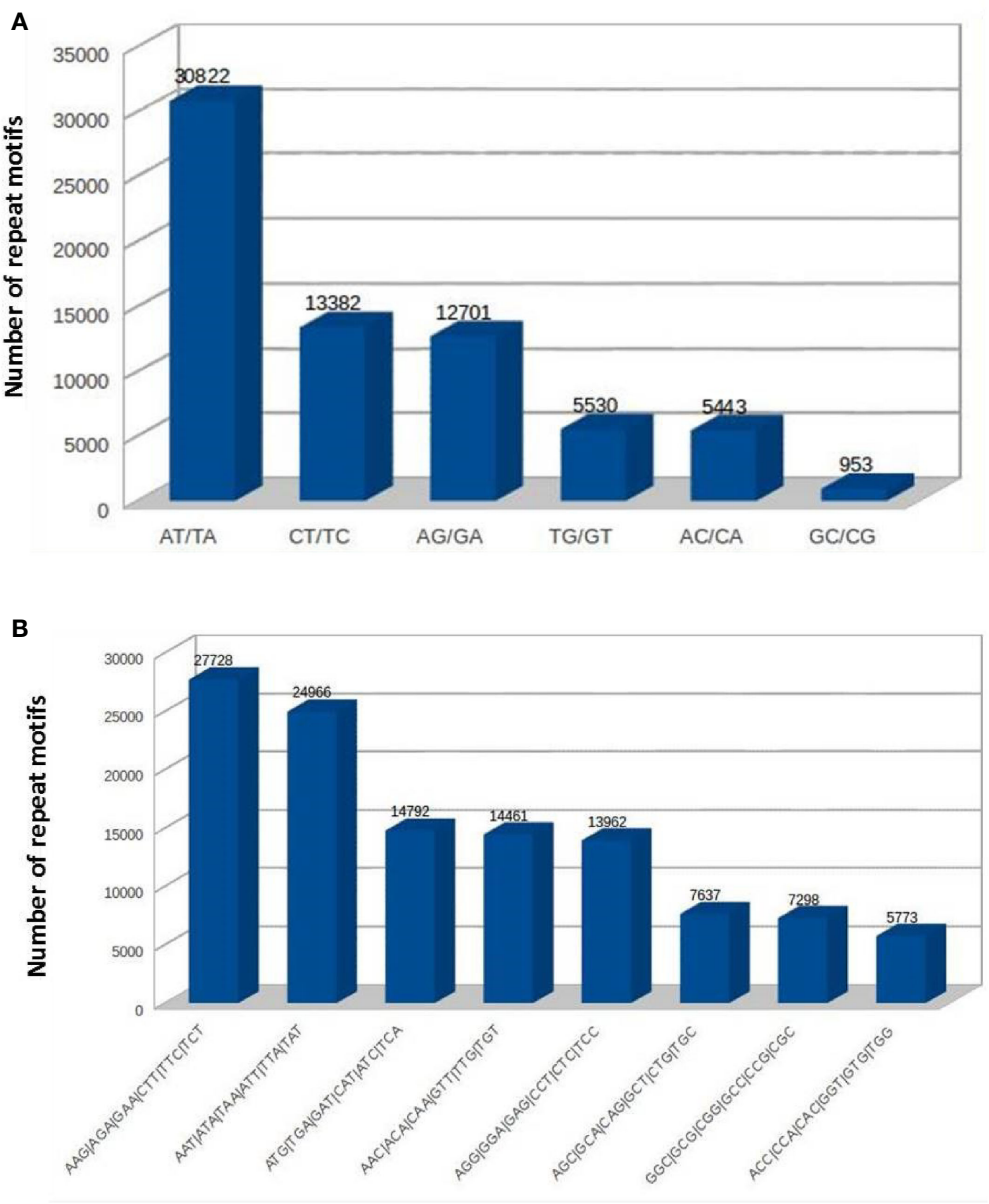
The frequencies of SSRs with different repeat sequence motifs in cardamom genome **(A)** dinucleotides. **(B)** trinucleotides.

The SSRs (dinucleotides to hexanucleotides) were identified from the assembled contigs. A total 2,50,571 SSRs were identified. Among these, 2,18,270 (87%) were perfect and 32,301 (12%) were compound SSRs. Among the perfect SSRs, trinucleotides were most abundant (1,25,329), followed by dinucleotides (68,831), tetranucleotides (16,794) and pentanucleotides (4936). The hexanucleotide repeats were least in number (2,380) (**Figure 3**). The repeat motif (AT/TA) was highest in number among the dinucleotides (**Figure 4A**) and the repeat motifs (AAG/AGA/GAA/CTT/TTC/TCT) were the most abundant among trinucleotides (**Figure 4B**). Our results are in agreement with the previous studies where dinucleotide repeats have been reported as the most abundant repeat type in crops like *Oryza sativa* (McCouch et al., 2002), mung bean (Tangphatsornruang et al., 2009), cranberry (Zhu et al., 2012), pigeonpea (Dutta et al., 2011), black alder (Lepais and Bacles, 2011), maqui (Bastías et al., 2016) and black pepper (Kumari et al., 2019). In some crops trinucleotides were the most abundant type of SSRs like in *Glycine max* (Xin et al., 2012), *Brachypodium* (Sonah et al., 2011), foxtail millet (Zhang et al., 2014) and watermelon (Zhu et al., 2016). In cotton, however hexanucleotides were the most abundant SSRs in *Gossypium hirsutum* and pentanucleotides in *G. raimondii* genome (Wang et al., 2015). The differences may also be due to use of different software programs and also the input parameters used for identification of SSRs and the completeness of the genome assemblies used for prediction.

### Development and validation of SSR markers

**Figure 5:**
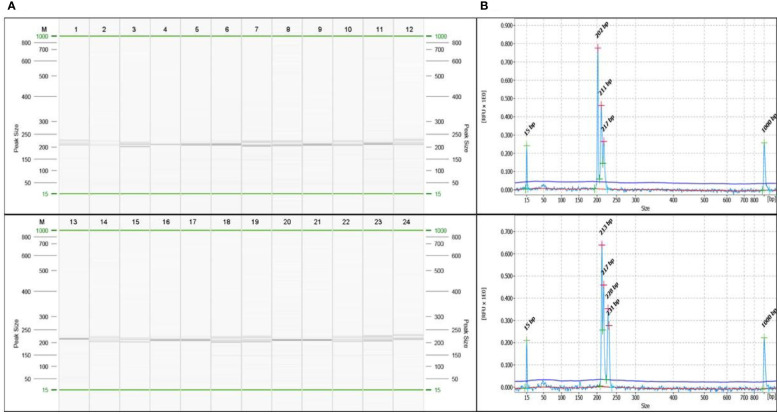
**(A)** A representative profile of amplification at locus EC86 in 24 accessions of cardamom captured on QIAxcel ScreenGel software. The lane marked ‘M’ is DNA molecular weight standard 50-800 bp v2.0 Qx DNA size marker. **(B)** A representative electropherogram showing different allele sizes in sample 3 and sample 24 for the marker EC86.

SSR markers play an important role in genetic diversity analysis, gene tagging, mapping and marker assisted selection. In earlier studies, the SSR markers from related genera like *Amomum subulatum*, *Zingiber officinale* and *Curcuma longa* were tested for transferability in cardamom (Anjali et al., 2015; Cyriac et al., 2015; Sakthipriya and Sabu, 2018). The present report of genomic SSR markers in small cardamom paves the way for further downstream applications like genetic diversity analysis, QTL mapping, marker assisted selection etc. The flanking sequences of SSRs identified from whole genome sequences of cardamom were used for designing primers. A total of 2,27,808 primers were designed from 2,50,571 SSRs mined. For wet lab validation, 246 primers were synthesized and tested for amplification in three cardamom genotypes. Out of 246 primer pairs, 136 produced amplification product of expected size of which 60 were further used for diversity analysis in a set of 60 cardamom accessions. A representative amplification profile of 24 cardamom accessions with SSR primer EC 86 as resolved on QIAxcel multi-capillary system is shown in **Figure 5**.

### Genetic diversity analysis

**Figure 6:**
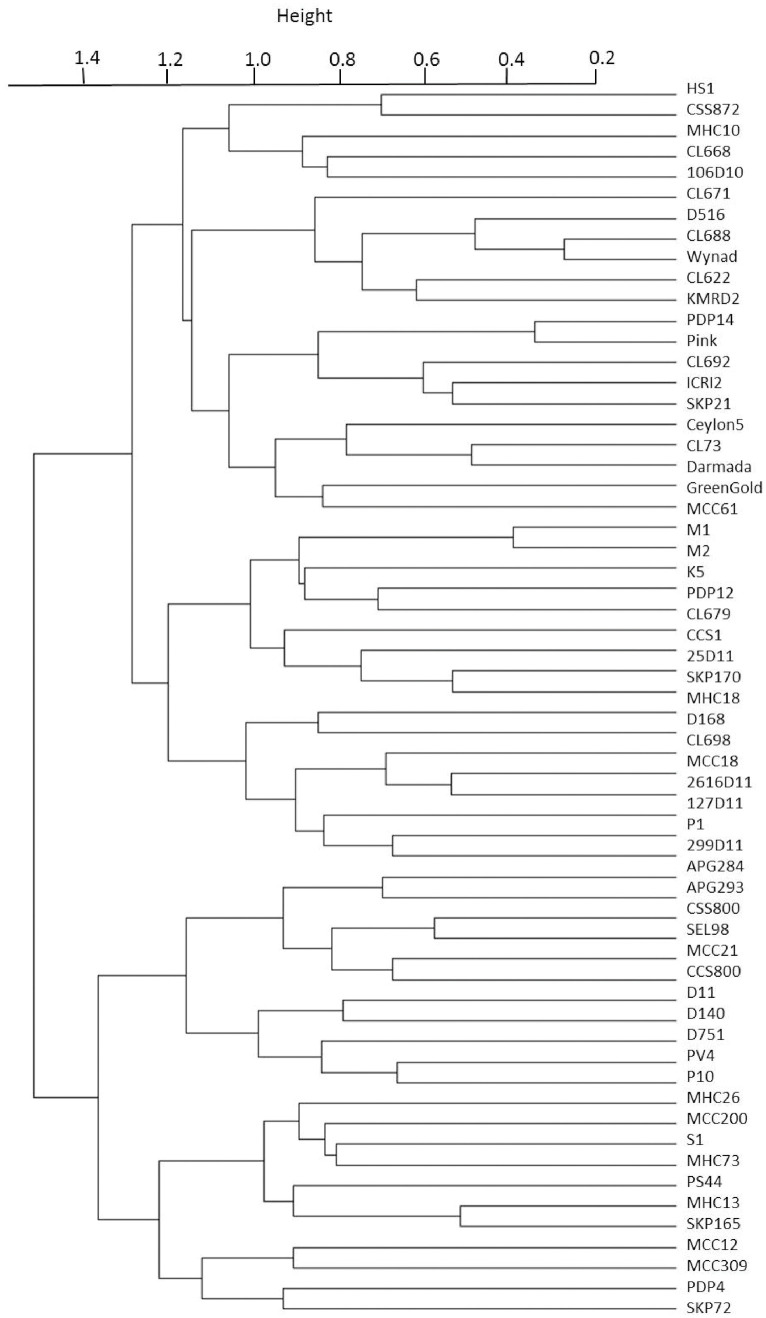
Hierarchical clustering of 60 cardamom accessions at 60 SSR loci based on Bruvo distance.

From 60 primers, a total of 874 alleles were detected with an average of 14.57 alleles per locus. The number of alleles ranged from a minimum of 4 to a maximum of 30. The marker EC 49 had the highest value for polymorphism information content (PIC) i.e., 0.80 while the marker EC 12 had the least PIC value of 0.08. We identified a set of SSR primers with high PIC (> 0.6) i.e., EC 1, EC 33, EC 47, EC 89, EC 90, EC 106, EC 183, EC 193, EC 196 and EC 212. Based on diversity analysis, the 60 accessions were clustered into two different groups which were each further divided into two smaller groups (**Figure 6**). The average genetic distance between the accessions was estimated to be 0.61 with highest distance of 0.75 between the two accessions 29-9-D11 and MCC12 and least distance of 0.17 between the cardamom accessions, Wayanad and CL-688. Compared to the previously reported ISSR (Anjali et al., 2016) and SSR (Cyriac et al., 2016) markers in cardamom, the average number of polymorphic bands generated by the SSR markers identified in the present study was much higher. The number of alleles detected by the use of ISSRs and SSRs ranged from 2 to 7 in the previous studies. Here, we obtained substantially higher number of alleles with a comparable number of cardamom accessions used. This implies that the SSR markers identified and used in the present study have significantly higher discriminatory powers and can be efficiently utilized for germplasm characterization. The availability of highly polymorphic SSR markers would also allow identification and mapping of genomic loci governing superior agronomic traits in cardamom.

### Population structure

**Figure 7:**
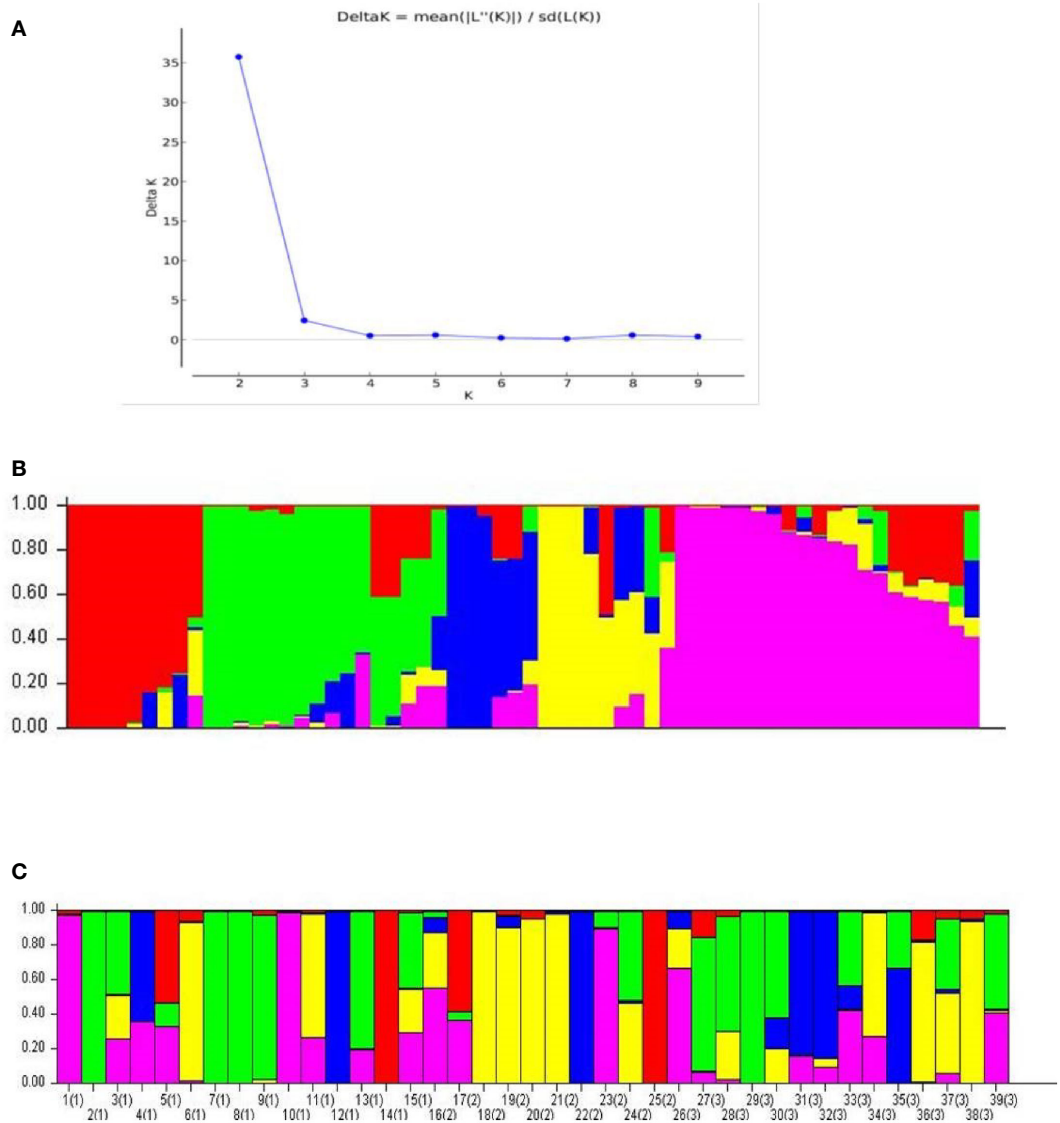
Population structure analysis for the 60 cardamom accessions. **(A)** Delta *K* (Δ*K*) plot from Structure Harvester for estimation of different numbers of subpopulations. **(B)** Population structure of 60 cardamom accessions with *K* = 5. **(C)** Population structure for the 39 different accessions of cardamom belonging to different panicle types i.e., Malabar (prostrate), Mysore (erect) and Vazhukka (intermediate or semi-erect) types.

The Structure analysis revealed presence of five groups among the 60 accessions of cardamom (**Figures 7A, B**). For the 39 accessions, which were categorized as the Malabar (prostrate panicle, group 1), Mysore (erect panicle, group 2) and Vazhukka (intermediate or semi-erect panicle, group 3) types, the proportion of membership of each panicle type groups in each of the five clusters is indicated in **Table 6**. The inferred ancestry of these accessions in the five clusters is given in **Supplementary Figure 3**. The group 1 cultivars were distributed in all five clusters although the clusters 2 and 5 had major proportion. However, cultivars from group 2 were mainly placed in cluster 4 with sizeable number in cluster 3 and 5. The pattern of grouping for group 3 cultivars was mostly comparable to that for group 1 cultivars, with greater representations in clusters 2, 3, 4 and 5 which is explainable since these are cultivars with intermediate type panicles. This was also supported by the mean values of Fst (pairwise fixation statistic) for the five groups indicating further the lack of genetic differentiation of cultivars from different panicle type groups (**Supplementary Table 5**). Similar observations have been made previously for the cardamom accessions with different panicle types (Prasath and Venugopal, 2004). No well-defined clustering was observed for the Malabar, Mysore and Vazhukka panicle type accessions. The possibility of existence of a common ancestral origin might be a reason for the lack of genetic differentiation observed. These inferences were further substantiated by the bar diagrams of genotypic constitutions of the cultivars analysed (**Figure 7C**). The cultivar-wise bar plots indicated presence of high degree of genetic admixtures in all panicle type groups. The bar plot indicated presence of 13 cultivars with least or no admixture of alleles from other groups (1-CL692, 2-CL688, 7-M1, 8-M2, 9-HS1, 10-SKP21, 12-CCS800, 14-SKP165, 18-Darmada, 21-MCC-12, 22-MCC21, 25-MHC13, 29-D516 and 38-MCC309). The presence of large-scale admixtures due to predominant cross-pollinations prevailing in this species appears to have resulted in lack of population differentiation among the panicle type groups of cardamom. However, lack of admixture in the 13 cultivars listed above needs to be related to their significance to agronomic performance, yield, adaptability, propagation procedures and their pedigree.

**Table 6:** Proportion of membership of each of the pre-defined populations in the five clusters.

Given pop	Inferred Clusters					Number of individuals
	1	2	3	4	5	
1 Malabar (prostrate)	0.111	0.388	0.111	0.143	0.248	15
2 Mysore (erect)	0.165	0.072	0.118	0.462	0.183	10
3Vazhukka (semi-erect)	0.034	0.342	0.201	0.265	0.158	14

### Green cardamom db

The green cardamom db is a freely available public database which can be utilized to search for microsatellite repeats and their corresponding primers for PCR based amplification of SSR markers at the 2,27,808 loci in cardamom.The database provides a SSR search option under the Microsatellite tab. Genomic SSRs can be searched based on the length and the sequence of the core motif. This database would serve as an important repository for information regarding SSR markers developed for cardamom and facilitate comparative and functional genomics studies for cardamom in the future (http://www.nbpgr.ernet.in:9092/).

## Conclusion

The WGS of cardamom was assembled *de novo* and the sequence length of the assembled genome was 1.06 Gb. More than 75% of the genome was sequenced through a hybrid sequencing strategy which involved three different sequencing chemistries i.e., the Oxford Nanopore, Illumina and 10x Genomics GemCode. The genome appears to be very rich in repeat regions and 68,055 gene models were predicted. At its current assembly levels, it appears very close to the members of the *Musa* family. The sequence information generated was utilized for mining and characterization of SSRs. A total of 2,50,571 SSRs were identified across the genome of cardamom and about sixty SSR markers were used for diversity analysis for a set of sixty cardamom accessions collected on the basis of their morphology and distribution in the native growing areas. These markers were highly polymorphic with an average of 14.57 alleles amplified across the cardamom accessions. The population structure analysis revealed the presence of high degree of genetic admixtures due to prevalent cross-pollinations in the species. The availability of SSR markers providing genome-wide coverage would find immense utility in future studies aimed at crop improvement through marker –assisted breeding in cardamom. Through this study, we have developed novel genomic resources in cardamom which were hitherto not reported.

## Data availability statement

The datasets presented in this study can be found in online repositories. The names of the repository/repositories and accession number(s) can be found in the article/**Supplementary Material**.

## Author contributions

Conceptualization and funding acquisition (AG and KB); Investigation and data analysis (AG, PR, RK, KB); Manuscript preparation (SY, AG, DW). All authors contributed to the article and approved the submitted version.
