# Supplementary material for: Small cardamom genome: development and utilization of microsatellite markers from a draft genome sequence of Elettaria cardamomum Maton
Source: Front Plant Sci. 2023 May 10;14:1161499. doi: 10.3389/fpls.2023.1161499 (PMC10206324; doi:10.3389/fpls.2023.1161499)

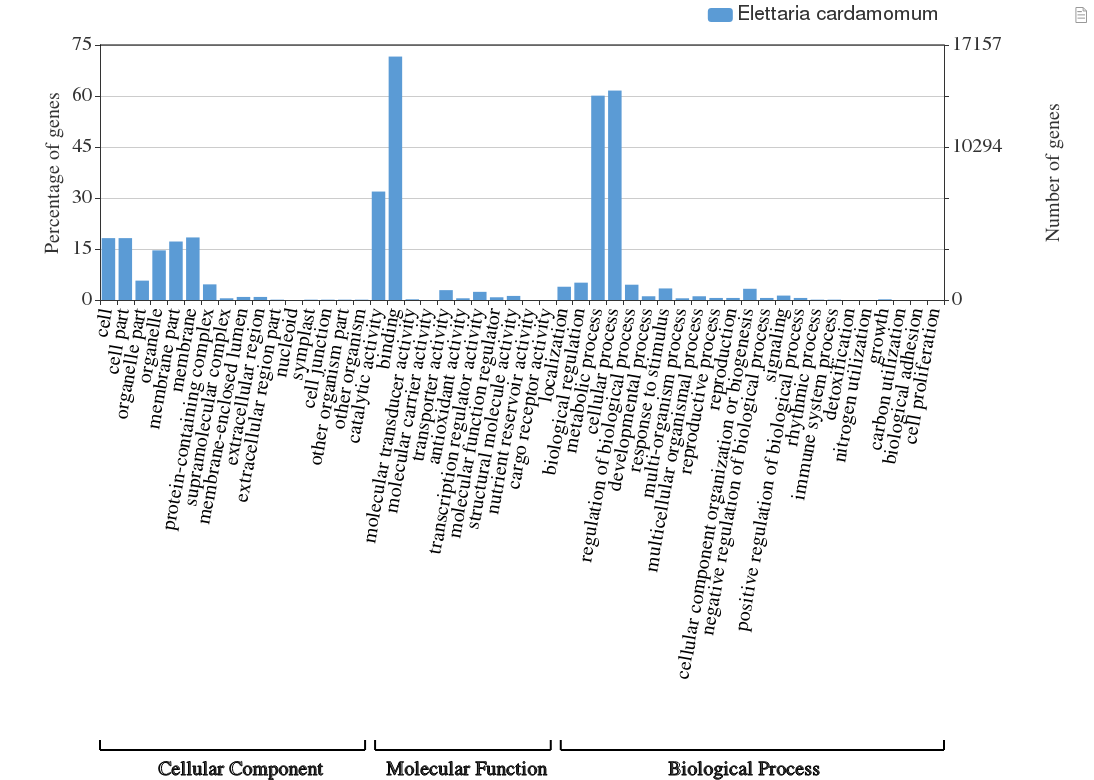


# SUPPLEMENTARY FIGURE 1 Gene ontology (GO) enrichment analysis for the genes in terms of the enrichment for the three GO terms; cellular component, molecular function and biological process.


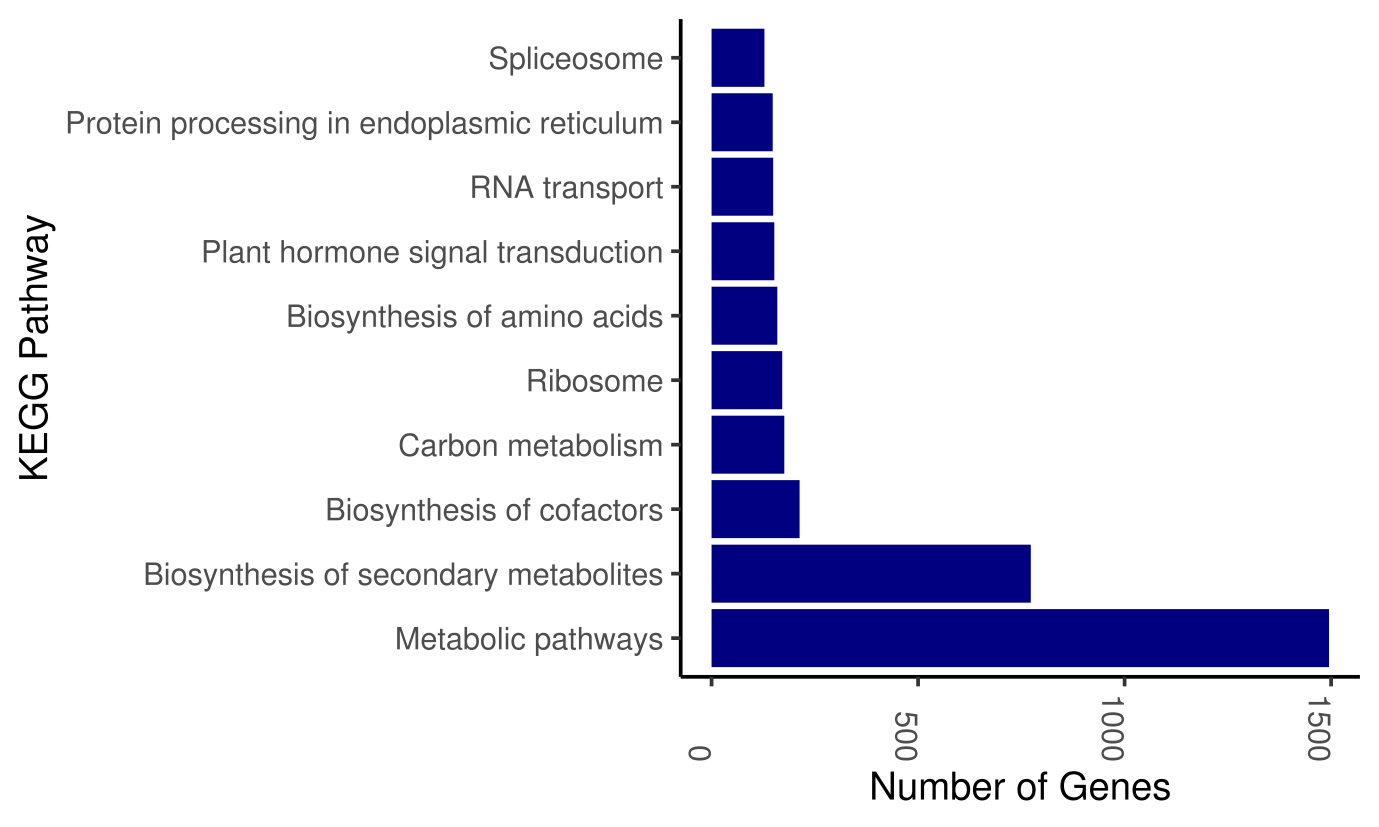


**SUPPLEMENTARY FIGURE 2** Kyoto Encyclopedia of Genes and Genomes (KEGG) enrichment analysis for the cardamom genes.

**SUPPLEMENTARY FIGURE 3** The inferred ancestry of 39 accessions (with different panicle types ) in the five clusters (k=5).


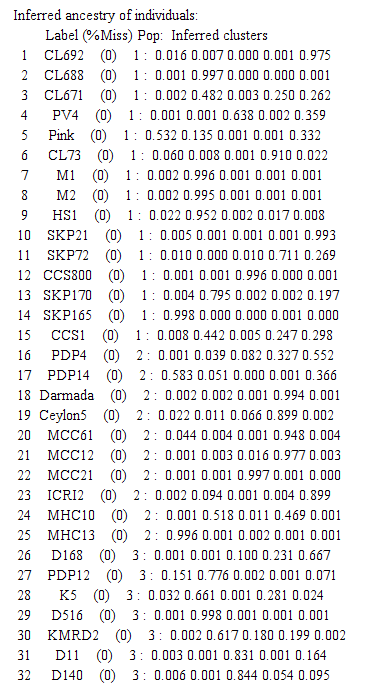


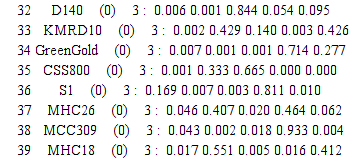

Supplement: Supplementary file 1 [file DataSheet_1.docx]
